# Regenerative Endodontic Therapy in the Management of Immature Necrotic Permanent Dentition: A Systematic Review

**DOI:** 10.1155/2020/7954357

**Published:** 2020-07-13

**Authors:** Faisal T. Alghamdi, Alaa E. Alqurashi

**Affiliations:** ^1^Department of Oral Biology, Faculty of Dentistry, King AbdulAziz University, Jeddah 80209, Saudi Arabia; ^2^Ibn Sina National College for Medical Studies, Dental College, Jeddah 22421, Saudi Arabia

## Abstract

**Materials and Methods:**

The electronic databases PubMed and Google Scholar were used to search the literature for relevant studies after applying specific inclusion and exclusion criteria. Studies that fulfilled both the inclusion and exclusion criteria were included in this systematic review. The search was conducted by two independent reviewers following the PRISMA guidelines.

**Results:**

Only 46 studies that fulfilled both the inclusion and exclusion criteria, which were conducted within the last 10 years, were included in this systematic review. These studies investigated different aspects of regenerative endodontic therapy including different types of scaffolds, intracanal medications, pulpal space/barriers, root maturation stage, follow-up duration, and updated studies on their use in the management of immature necrotic permanent teeth.

**Conclusions:**

This review concluded the compiled data observed that endodontic regenerative therapy was more efficient in treating immature necrotic permanent teeth and offered a greater advantage that should lead to wider acceptance among endodontists for effective results compared to different treatment options. However, more clinical trials with a standardized protocol and defined clinical, radiographic, and histopathological outcomes with longer follow-up periods are warranted.

## 1. Introduction

Regenerative endodontic treatment (RET) is a common category of biologically based endodontic therapy known as revascularization or revitalization; RET aims to promote normal physiological development in immature permanent teeth with pulpal necrosis [1, 2]. Resolution of apical periodontitis, retrogression of associated clinical symptoms, and prolonged survival of teeth are other important outcomes of RET [2–4], which are favored in children and young individuals as a viable alternative to traditional (nonregenerative) endodontic procedures [2].

In regard to immature necrotic permanent teeth, there are many treatment options for immature nonvital teeth, including periapical surgery and apexification using calcium hydroxide (Ca(OH)_2_) and mineral trioxide aggregate (MTA) or biodentin [5–7]. The apexification was used as a treatment option for many years, which induces the formation of a calcific barrier at the apex by using intracanal calcium hydroxide (Ca(OH)_2_). In spite of the extensive use of the Ca(OH)_2_-based apexification procedure, the long procedure time might require several visits and reestablishment of the intracanal dressing [8, 9], arbitrariness of the apical closure [10], and the predisposition of cervical root fractures after extended exposure to Ca(OH)_2_ [11] have raised earnest disquiet about the eligibility of this procedure technique. Up-to-date, the conventional apexification technique has been adjusted by the presentation of artificial apical barrier methods with mineral trioxide aggregate (MTA) [12–14]. The MTA approach enhances patient compliance and good outcomes to assist in the healing of the periapical tissues, although a shorten treatment period [12, 13, 15, 16]; on the other hand, unfortunately, the improvement of the apical closure and intensification of radicular dentin still cannot be achieved by this approach [10, 11]. Based on these considerations, it may be the apexification in future treatment protocols for nonvital immature permanent teeth illustrates to be dubitable [17, 18].

The idea of endodontic regeneration as a treatment option was favored especially after two authors in 2004; they illustrated a new technique for the management of immature permanent teeth with apical periodontitis and termed it “revascularization” [19]. The endodontic society found that “Pulp revascularization” was a significant advance to investigate tracks of pulp and dentin regeneration [20, 21]. Revascularization depends on the stem cells and growth factors by stimulating them to complete the apex closure. It is widely used when the opening diameter of the root canal is large [22]. Moreover, revascularization treatment enhanced root elongation and maturation [23].

There are many investigations which confirmed the success rate of the regenerative endodontic procedure conducted on children and young individuals, but the present evidence is still disputable in regards to the regenerated tissue and regenerative protocol. Few systematic review studies are available for the management of these teeth by regenerative endodontic treatment. Therefore, this current systematic review aims to compile all up-to-date information that investigated endodontic regeneration therapy in the management of immature permanent teeth with necrotic pulp and which conducts are most used and appropriate for this procedure in human and animal investigations.

## 2. Materials and Methods

This systematic review was designed and executed under the PRISMA guidelines [24].

### 2.1. Literature Search Strategy

Bibliographical searches were carried out in PubMed and Google Scholar databases in December 2019 and then updated in May 2020, using the Mesh terms, which were combined with Boolean operators (“AND” and “OR”). The following search strategies were used: “immature teeth” OR “immature tooth” OR “immature dentition” OR “immature permanent teeth” OR “immature permanent tooth,” AND “young permanent teeth” OR “young permanent tooth,” AND “pulp revascularization” OR “pulpal regeneration” OR “pulp revitalization” OR “root canal revascularization” OR “root maturation” OR “regenerative endodontic” OR “regenerative endodontic therapy” OR “regenerative endodontic treatment” OR “regenerative endodontic procedure,” AND “blood clot” OR “platelet-rich fibrin” OR “platelet-rich plasma,” AND “calcified barrier” OR “apical closure” OR “root end formation” OR “root apex closure” OR “apical plug” OR “MTA plug” OR “apexification” OR “mineral trioxide aggregate” OR “calcium hydroxide.” The search database was examined by both examiners, and the final decision for inclusion and exclusion was made according to the following criteria. Studies that meet the following inclusion criteria were considered eligible: (1) published studies between the 10 years (2009–2019); (2) original research articles in the English language; and (3) studies performed on human and animal subjects. The following were considered as exclusion criteria: (1) published studies that assessed regenerative endodontic therapy but excluded immature necrotic permanent teeth; (2) studies that discuss the management of immature necrotic permanent teeth but excluded their effect on root closure or development; and (3) review articles on the management of immature nonvital permanent teeth.

### 2.2. Critical Appraisal

All reviewers independently screened the titles and abstracts of retrieved articles according to the eligibility criteria as well as PRISMA guidelines. Disagreements or inconsistencies were resolved through discussion and consensus among the two reviewers.

### 2.3. Data Extraction

The data were checked for completeness, accuracy, and extracted into standardized Microsoft Office Excel worksheets by both reviewers on an independent basis by fully reading the articles and considering the following variables: title, abstract, material and methods (number of subjects (teeth), type of intracanal medication, scaffolds, pulpal space/barrier, root maturation stage, and follow-up duration), and main results.

### 2.4. Data Items

Data from the included articles were collected and organized in columns as the following:

#### 2.4.1. Human Studies

These studies include the following information: author and year, age of the patient with mean and standard deviation, number of subjects (teeth), type of intracanal medication, scaffolds, pulpal space/barrier, root maturation stage, follow-up duration, and main outcomes.

#### 2.4.2. Animal Studies

These studies include the following information: author and year, animal species, number of subjects (teeth), type of intracanal medication, scaffolds, pulpal space/barrier, root maturation stage, follow-up duration, and main outcomes.

### 2.5. Assessment of Methodological Quality

As part of the data extraction process, two review authors assessed the risk of bias of the included studies. The methodological quality of each study was performed by using the risk of a bias assessment tool outlined in the Cochrane Handbook for Systematic Reviews of Interventions (Version 5.1.0) [25].

### 2.6. Synthesis of Results

As mentioned, tables were prepared with the fields included as data items.

### 2.7. Statistical Analysis

Parametric data involving the age of the patients of the human studies are presented as mean and standard deviation (M ± SD). Thus, only a descriptive evaluation is presented.

## 3. Results and Discussion

### 3.1. Results

#### 3.1.1. Study Selection

**Figure 1:**
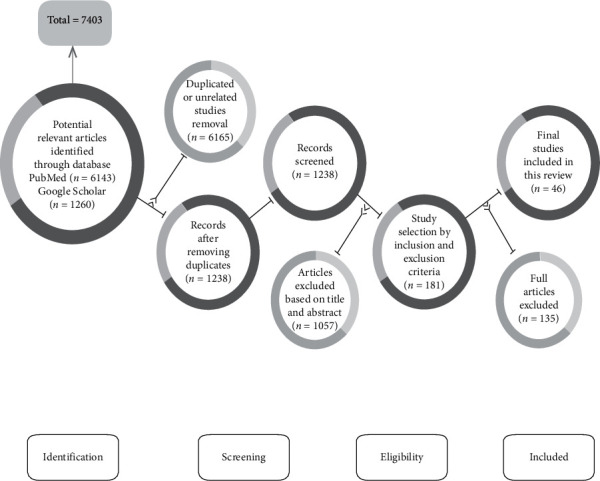
Flowchart outlining the protocol adopted in the systematic review based on the preferred reporting items for systematic reviews and meta-analyses (PRISMA) guidelines.

Among the 7403 articles selected through the keywords using the databases, duplicated or unrelated records (*N* = 6165) were excluded; only 181 articles were initially listed according to the inclusion and exclusion criteria. Finally, 46 articles were selected to include in this systematic review. The summary of the selection process of the articles in this systematic review is delineated in Figure 1.

#### 3.1.2. Study Characteristics

The search culminated in forty-six studies that fulfilled both the inclusion and exclusion criteria. The review included randomized controlled trials, controlled clinical trials, case reports, in vitro with in vivo studies, in vivo studies, and prospective/retrospective studies comparing the effectiveness of pulp revascularization in immature necrotic permanent teeth [3, 4, 6, 16, 20, 22, 26–65]. The studies included in this systematic review were 31 human studies [3, 4, 6, 16, 20, 22, 26–31, 35, 39, 40, 44–47, 50–53, 55–57, 59, 60, 63–65] and 15 animal studies [32–34, 36–38, 41–43, 48, 49, 54, 58, 61, 62]. This systematic review evaluated 46 studies that included a study sample of 1006 subjects (teeth). The human studies included patients of children and young individuals (aged between 7 and 18 years) with a mean age (mean ± SD) 10.1 ± 3.18 years and selected from different dental clinics, hospitals, and dental schools [3, 4, 6, 16, 20, 22, 26–31, 35, 39, 40, 44–47, 50–53, 55–57, 59, 60, 63–65]. On the other hand, most of the samples in animal studies included different species such as ferrets, sheep, dogs, and monkeys [32–34, 36–38, 41–43, 48, 49, 54, 58, 61, 62]. Among the included studies, six studies illustrated negative outcomes in regard to endodontic regeneration therapy in the management of immature necrotic permanent teeth [29, 34, 46, 48, 49, 54]. On the other hand, 40 studies showed significant positive outcomes for endodontic regeneration treatment in these kinds of teeth due to root development, root wall thickening, root lengthening, and formation of hard tissue barrier or apical closure [3, 4, 6, 16, 20, 22, 26–28, 30–33, 35–45, 47, 50–53, 55–65]. The outcomes of these 46 studies include different types of intracanal medications, scaffolds, pulpal space/barrier, root maturation stage, follow-up duration, and updating studies on their effect in periapical periodontitis and periapical healing.  Table 1 provides a summary of the included human studies in this systematic review. An informative description of the included animal studies and their main outcomes are summarized in Table 2.

#### 3.1.3. Quality and Risk Assessment of the Included Studies

The quality and risk assessment of all the included studies were conducted by two authors and are represented in Table 3. Included studies were assessed following the Cochrane collaboration's tool [25] for assessing the risk of bias. Summarizing, no single study was classified as a high risk of bias, and most studies demonstrated low or unclear risk of bias (Table 3).

### 3.2. Discussion

This systematic review was conducted to summarize and appraise all appreciated studies published within the last 10 years and fulfilled our study aim. This current systematic review aimed to compile all up-to-date information that investigated endodontic regeneration therapy in the management of immature permanent teeth with necrotic pulp and which conducts are most used and appropriate for this procedure in human and animal investigations. Our study presents a comprehensive compilation of evidence taken from 46 articles that met our inclusion and exclusion criteria.

Up-to-date, we can only find two old systematic reviews that talked about endodontic regenerative therapy in the management of immature necrotic teeth for human and animal studies (Table 4) [66, 67]. Bucchi et al. concluded in their systematic review that most of the retrieved studies about clinical protocols of endodontic regenerative treatment suggest their effectiveness in the management of these kinds of teeth, however, most of the studies were found to support specific irrigation and intracanal dressings to better clinical, histological, and radiographic outcomes in endodontic regeneration for clinical human and animal studies [66]. In contrast, although Antunes et al. focused only on 11 articles in regards to pulp revascularization, their results confirm the clinical success of this procedure. In addition, the ability of this procedure is to activate the apical closure formation and increase the thickening of radicular dentin, but the key factors of tissue repair, the type of tissue formed, and the long-term prognosis are still not clear in different clinical studies [67]. This clearly shows the discrepancies in the conclusions between the previously published systematic reviewers. This may be mainly due to the differences in the applied inclusion and exclusion criteria in addition to the authors' opinions. However, we could find some studies to support the high success rates for the use of endodontic regenerative therapy in the management of immature necrotic permanent teeth in human and animal studies compared with previously published systematic reviews. In addition, the first review [66] covered the period time from 2007 to 2016 and the second review [67] covered the period time from 2008 to 2014 (Table 4); thus, our systematic review covered all eligible articles published within the last decade ((Table 1) and (Table 2)).

In our updated systematic review, all 46 studies favored the use of different scaffolds in endodontic regenerative therapy in the management of immature necrotic permanent teeth ((Table 1) and (Table 2)). The majority of these studies used blood clot (induced bleeding) as a scaffold in this procedure ((Table 1) and (Table 2)). Hence, the three scaffolds such as blood clot (induced bleeding), platelet-rich plasma (PRP), and platelet rich fibrin (PRF) have a vital role to stimulate pulp revascularization and associate to the treatment of immature necrotic permanent teeth [68]. In regards to the different types of intracanal medications and pulpal space/barriers used in this procedure, most of the investigations in this review used triple-antibiotics paste (TAP) as an intracanal medication and mineral trioxide aggregate (MTA) as the pulpal space/barrier due to their effectiveness in pulp revascularization to treat the immature necrotic permanent teeth in comparison with other intracanal medications and pulpal space/barriers ((Table 1) and (Table 2)). Most of the studies included in this review do not use any classification system to determine the degree of root formation and maturation. Each study has measured the percentage of root length and width changes to determine root maturation ((Table 1) and (Table 2)). In our review, we used a specific classification system to evaluate the degree of root formation and maturation based on these root length and width changes among the included studies ((Table 1) and (Table 2)). This classification is called “Cvek's Classification,” concerning root formation and maturation [69]. The Cvek's classification system was used in this systematic review due to didactic radiographic characteristics of this system, which allow for a better clinical application than that used in the other classification schemes [69]. This Cvek's classification system was used to determine the root maturation stage in the following five stages concerning the level of root maturity: stage I = less than 1/2 root length, stage II = 1/2 root length, stage III = 2/3 root length, stage IV = wide open apical foramen and nearly complete root length, and stage V = closed apical foramen and completed root development. Moreover, the teeth in stage V were considered mature and fully apical formed teeth, and the other four stages (stages I, II, III, and IV) described teeth with open apices and lack of apical constriction development but with some significant morphological differences [69]. Among the 31 human studies in this review, 26 studies showed partially or completely mature teeth (Cvek stages IV and V), 3 studies showed teeth with intermediate root development (Cvek stage III), and 2 studies showed teeth with an initial stage of root development (Cvek stages I and II) (Table 1). In contrast, among the 15 animal studies, 10 studies were classified as Cvek stages IV and V, followed by 1 study classified as Cvek stage III, 3 studies showed teeth with an initial stage of root development (Cvek stages I and II), and one study was not reported in regard to the root maturation stage (Table 2). Therefore, regenerative endodontic therapy has the potential to induce the root maturation of necrotic immature permanent teeth and illustrate a significant increase in root length and dentinal wall thickness in most of the included studies in this review ((Table 1) and (Table 2)). Although all these studies were carried out on human and animal models, we need more studies to be conducted to strengthen the evidence of these studies; thus, we will be very close to finally give this procedure the superiority in treating immature necrotic permanent teeth compared with other treatment options.

Studies included in this review illustrated varied follow-up times for endodontic regeneration therapy in the management of these kinds of teeth ((Table 1) and (Table 2)). Interestingly, only 6 studies reported negative outcomes related to the clinical outcomes of endodontic regeneration therapy in the management of these teeth [29, 34, 46, 48, 49, 54]. Although the results obtained from the included studies in this review confirm the success of this procedure in the management of immature necrotic permanent teeth in 40 studies ((Table 1) and (Table 2)). This warrants future randomized controlled clinical trials that scrutinize each clinical protocol with long-term outcomes of endodontic regenerative therapy and its clinical success in the management of these teeth.

## 4. Conclusions

This systematic review concluded that endodontic regenerative therapy showed better results in certain parameters such as increase in root wall lengthening and thickening, acute/chronic periapical lesions healing, and improved apical closure formation in the management of immature necrotic permanent teeth. In addition, there was considerable homogeneity among the included studies; so, conclusive results suggestive of their evidence towards the superiority of endodontic regenerative therapy in treating these kinds of teeth were compared with other treatment options. However, more clinical trials with a standardized protocol and defined clinical, radiographic, and histopathological outcomes with longer follow-up periods are warranted.

## Figures and Tables

**Table 1:** Summary of the included human studies in this systematic review according to PRISMA guidelines.

Authors (year)	No. of subjects (teeth)	Age of patient-years (mean ± SD)	Intracanal medications	Scaffolds	Pulpal space/barrier used	Follow-up duration	Root maturation stage (Cvek's classification)	Main outcomes
Alasqah et al. [26] (2020)	(*n* = 1)	8 years old	Ca(OH)_2_ + TAP	Blood clot	MTA plug	2 years	Stage V (closed apical foramen and completed root development)	Significant periapical healing and complete roots formation
Rizk et al. [27] (2019)	(*n* = 30)	(9.1 ± 1.02)	TAP	Blood clot + PRP	MTA	1 year	Stage V (closed apical foramen and completed root development)	Complete maturation of the root apex
Ajram et al. [28] (2019)	(*n* = 1)	7 years old	Ca(OH)_2_	Blood clot	Micromega-MTA (MM-MTA)	2 years	Stage V (closed apical foramen and completed root development)	Apical closure and complete periapical healing
Ulusoy and Cehreli [29] (2017)	(*n* = 4)	(9.2 ± 1.75)	TAP	Blood clot	MTA	1.5 years	Stage I (<1/2 root length with open apex)	Lack of evidence for increased root dimensions and/or apical closure, but the elimination of clinical signs/symptoms and resolution of apical periodontitis have happened.
Moodley et al. [30] (2017)	(*n* = 1)	10 years old	Ca(OH)_2_ + TAP	Blood clot	MTA	2–5 months	Stage V (closed apical foramen and completed root development)	Apical closure and a thickening of the dentinal walls
Timmerman and Parashos [31] (2017)	(*n* = 1)	16 years old	Ca(OH)_2_	Blood clot	MTA	3 years	Stage V (closed apical foramen and completed root development)	Root development/Apical closure
Nosrat et al. [35] (2015)	(*n* = 2)	(9.5 ± 0.74)	TAP	Blood clot	MTA	4 months	Stage V (closed apical foramen and completed root development)	Root development/Apical closure
Bezgin et al. [39] (2015)	(*n* = 20)	(9.9 ± 1.9)	TAP	Blood clot + PRP	MTA	1.5 years	Stage V (closed apical foramen and completed root development)	Complete apical closure, periapical tissue pathology resolution
Narang et al. [40] (2015)	(*n* = 20)	(11.2 ± 3.51)	TAP	Blood clot + PRP + PRF	Resin-modified glass ionomer cement	6 months–1.5 years	Stage V (closed apical foramen and completed root development)	Apical closure, root lengthening, dentinal wall thickening, and periapical healing
Saoud et al. [4] (2014)	(*n* = 20)	(11.3 ± 1.92)	TAP	Blood clot	MTA	1 year	Stage V (closed apical foramen and completed root development)	Apical closure, root lengthening, and root wall width
Alobaid et al. [44] (2014)	(*n* = 31)	(8.8 ± 1.67)	TAP + BAP + CH	Blood clot	MTA	8.5–14.5 months	Stage V (closed apical foramen and completed root development)	Apical closure and hard tissue barrier
Nagata et al. [45] (2014)	(*n* = 23)	(11.3 ± 3.12)	TAP + Ca(OH)_2_+ CH	Blood clot	MTA	9–19 months	Stage V (closed apical foramen and completed root development)	Root thickening/Lengthening/Apical closure
Kahler et al. [46] (2014)	(*n* = 16)	(10.1 ± 1.88)	TAP	Blood clot	MTA	1–3 years	Stage II (1/2 root length with open apex)	Root thickening/lengthening. Negative results for this procedure
Nagy et al. [47] (2014)	(*n* = 36)	(10.8 ± 1.54)	TAP	Blood clot + blood clot with (FGF)	MTA plug	1 years	Stage V (closed apical foramen and completed root development)	Root thickening/Lengthening/Apical closure
Jadhav et al. [50] (2013)	(*n* = 6)	(15.3 ± 6.82)	Ciprofloxacin + metronidazole + minocycline	Blood clot + blood clot with PRP	Resin-modified glass ionomer cement	1 year	Stage V (closed apical foramen and completed root development)	Root thickening/lengthening/Apical closure
Sönmez et al. [51] (2013)	(*n* = 3)	9 years old	Ciprofloxacin + metronidazole + minocycline	Blood clot	MTA	2 years	Stage V (closed apical foramen and completed root development)	Apical closure and dentin wall thickening
Mc Tigue et al. [52] (2013)	(*n* = 32)	(10.2 ± 1.83)	TAP	Blood clot	MTA	1 year	Stage V (closed apical foramen and completed root development)	Apical closure and root wall thickening + periapical tissue healing
Martin et al. [53] (2013)	(*n* = 1)	9 years old	TAP	PRP	MTA	1 year	Stage III (2/3 root length with open apex)	Root thickening/lengthening
Dabbagh et al. [55] (2012)	(*n* = 18)	(10.5 ± 1.58)	TAP	Blood clot	MTA	2 years	Stage IV (wide opening apical foramen and nearly completed root length)	Root thickening/lengthening, hard tissue barrier, and periapical tissue healing
Chen et al. [3] (2012)	(*n* = 20)	(10.2 ± 1.49)	Ca(OH)_2_	Blood clot	MTA	6–26 months	Stage IV (wide opening apical foramen and nearly completed root length)	Root lengthening, dentinal wall thickening, hard tissue barrier, and periapical healing
Jeeruphan et al. [6] (2012)	(*n* = 61)	(12.9 ± 5.07)	Ca(OH)_2_	Blood clot	Gutta-percha	11.7–21.15 months	Stage III (2/3 root length with open apex)	Root wall width/lengthening
Kim et al. [56] (2012)	(*n* = 3)	(10.6 ± 1.15)	Ciprofloxacin + metronidazole + cefaclor	Blood clot	MTA	2–4 years	Stage III (2/3 root length with open apex)	Periapical healing and dentin wall thickening
Iwaya et al. [57] (2011)	(*n* = 1)	7 years old	Ca(OH)_2_	Empty scaffold	Gutta-percha	30 months	Stage IV (wide opening apical foramen and nearly completed root length)	Continued root development and apical closure
Torabinejad and Turman [20] (2011)	(*n* = 1)	11 years old	TAP	Blood clot	MTA	5.5 months	Stage V (closed apical foramen and completed root development)	Hard tissue barrier
Cehreli et al. [16] (2011)	(*n* = 6)	10 years old	CH	Blood clot	MTA plug	1.5 years	Stage V (closed apical foramen and completed root development)	Apical closure, periapical tissue healing, and tissue regeneration
Nosrat et al. [59] (2011)	(*n* = 2)	(8.5 ± 0.70)	TAP	Blood clot	Calcium enriched mixture (CEM) cement	15–18 months	Stage IV (wide opining apical foramen and nearly completed root length)	Root development/Periapical tissue healing
Petrino et al. [60] (2010)	(*n* = 6)	(10 ± 3.60)	TAP	Blood clot	MTA	8 months	Stage V (closed apical foramen and completed root development)	Hard tissue barrier
Thomson and Kahler [63] (2010)	(*n* = 1)	12 years old	TAP	Blood clot	MTA	1.5 years	Stage IV (wide opening apical foramen and nearly completed root length)	Continued root development and some of the apical closures are evident
Reynolds et al. [22] (2009)	(*n* = 2)	11 years old	TAP	Blood clot	MTA	1.5 years	Stage V (closed apical foramen and completed root development)	Significant root development with maturation of the dentine
Ding et al. [64] (2009)	(*n* = 12)	(9.5 ± 1.16)	Ciprofloxacin + metronidazole + minocycline	Blood clot	MTA	15 months	Stage IV (wide opening apical foramen and nearly completed root length)	Continued root development
Bose et al. [65] (2009)	(*n* = 88)	Not reported	TAP + CH + formocresol	Blood clot	MTA	6 months-3 years	Stage IV (wide opening apical foramen and nearly completed root length)	Root development/thickening/lengthening

SD: standard deviation; Ca(OH)_2_: calcium hydroxide; TAP: triple-antibiotics paste; BAP: bi-antibiotics paste; CH: chlorhexidine; MTA: mineral trioxide aggregate; blood clot with bFGF: blood clot with basic fibroblast growth factor; PRP: platelet-rich plasma; PRF: platelet-rich fibrin; blood clot with FGF: blood clot with fibroblast growth factor; and DPCs: dental pulp cells. Gelfoam (Pfizer, New York, NY, USA).

**Table 2:** Summary of the included animal studies in this systematic review according to PRISMA guidelines.

Authors (year)	No. of subjects (teeth)	Animal species	Intracanal medications	Scaffolds	Pulpal space/barrier used	Follow-up duration	Root maturation stage (Cvek's classification)	Main outcomes
Bakhtiar et al. [32] (2017)	(*n* = 32)	Dogs	Ciprofloxacin + metronidazole + cefaclor	Treated dentine matrix (TDM) + tricalcium phosphate (TCP)	MTA	1 years	Stage V (closed apical foramen and completed root development)	Root development/Apical closure
Altaii et al. [33] (2017)	(*n* = 4)	Sheep	TAP	Blood clot	MTA	6 months	Stage V (closed apical foramen and completed root development)	Root development/Apical closure
Saoud et al. [34] (2015)	(*n* = 16)	Dogs	TAP	Blood clot	MTA	3 months	Not reported	Not reported in regards to the root development and apical closure, but there are significant results of thickening of the dentinal walls and periapical healing
Torabinejad et al. [36] (2015)	(*n* = 24)	Ferrets	TAP	Blood clot/Gelfoam + PRP + empty negative control	MTA	3 months	Stage IV (wide opening apical foramen and nearly completed root length)	Significantly more apical narrowing and hard tissue deposition in two scaffold groups compared with not using a scaffold
Londero Cde et al. [37] (2015)	(*n* = 30)	Dogs	TAP	Blood clot + blood clot with gelfoam + empty negative control	MTA	7 months	Stage V (closed apical foramen and completed root development)	Apical root development
Rodríguez-Benítez et al. [38] (2015)	(*n* = 40)	Dogs	Modified triantibiotic paste (mTAP)	Blood clot + PRP	Not report	6 months	Stage V (closed apical foramen and completed root development)	Root thickening/Apical closure
Khademi et al. [41] (2014)	(*n* = 36)	Dogs	TAP	Blood clot	MTA	3–6 months	Stage V (closed apical foramen and completed root development)	Apical closure, dentinal wall thickening, and periapical healing
Yoo et al. [42] (2014)	(*n* = 30)	Dogs	No report	Blood clot	MTA	12 weeks	Stage V (closed apical foramen and completed root development)	Apical closure and dentinal wall thickening
Zhang et al. [43] (2014)	(*n* = 27)	Dogs	TAP	Blood clot + PRP	MTA	3 months	Stage V (closed apical foramen and completed root development)	Apical closure and root wall thickening
Tawfik et al. [48] (2013)	(*n* = 108)	Dogs	TAP	Blood clot + blood clot with bFGF + empty negative control	MTA	1 week, 3 weeks, and 3 months	Stage I (<1/2 root length with open apex)	Negative results in this study. Root lengthening/thickness did not change.
Zhu et al. [49] (2013)	(*n* = 56)	Dogs	TAP	Blood clot + blood clot with DPCs + PRP + PRP with DPCs	MTA	3 months	Stage II (1/2 root length with open apex)	Root thickening only, and does not report about the apical closure
Petrović et al. [54] (2013)	(*n* = 24)	Monkeys	Not report	PRP with hydroxyapatite (HAP)	Glass ionomer cement (GIC) and amalgam	6 months	Stage I (<1/2 root length with open apex)	Retardation of root development and nonsignificant differences among the samples.
Yamauchi et al. [58] (2011)	(*n* = 64)	Dogs	TAP	Blood clot	MTA	3.5 months	Stage III (2/3 root length with open apex)	Periapical healing and root wall thickening
Zuong et al. [61] (2010)	(*n* = 6)	Dogs	TAP	Blood clot	MTA	8 weeks	Stage V (closed apical foramen and completed root development)	Apical closure/Root thickening
Da Silva et al. [62] (2010)	(*n* = 40)	Dogs	(TAP)	Empty scaffold	MTA	3 months	Stage IV (wide opening apical foramen and nearly completed root length)	Hard tissue barrier and increase of apical periodontal ligament thickness

Ca(OH)_2_: calcium hydroxide; TAP: triple-antibiotics paste; BAP: bi-antibiotics paste; CH: chlorhexidine; MTA: mineral trioxide aggregate; blood clot with bFGF: blood clot with basic fibroblast growth factor; PRP: platelet-rich plasma; PRF: platelet-rich fibrin; blood clot with FGF: blood clot with fibroblast growth factor; and DPCs: dental pulp cells. Gelfoam (Pfizer, New York, NY, USA).

**Table 3:** Quality and risk Assessment of all the included studies in this systematic review.

Study (year)	Random sequence generation	Allocation concealment	Defined inclusion/exclusion	Blinding of outcome assessment	Incomplete outcome data	Selective reporting	Other sources of bias
Alasqah et al. [26] (2020)	+	+	+	+	+	+	+
Rizk et al. [27] (2019)	+	+	+	+	+	?	+
Ajram et al. [28] (2019)	+	+	+	+	+	+	+
Ulusoy and Cehreli [29] (2017)	+	+	+	?	+	+	?
Moodley et al. [30] (2017)	?	+	+	+	+	+	+
Timmerman and Parashos [31] (2017)	+	+	+	+	+	+	+
Bakhtiar et al. [32] (2017)	+	+	+	+	+	+	+
Altaii et al. [33] (2017)	+	+	+	+	+	+	+
Saoud et al. [34] (2015)	+	+	+	+	?	+	+
Nosrat et al. [35] (2015)	?	?	+	+	+	+	+
Torabinejad et al. [36] (2015)	+	+	+	+	+	+	+
Londero Cde et al. [37] (2015)	+	+	+	+	+	?	?
Rodríguez-Benítez et al. [38] (2015)	+	+	+	+	+	+	+
Bezgin et al. [39] (2015)	+	+	+	+	+	+	+
Narang et al. [40] (2015)	+	+	+	+	+	+	+
Saoud et al. [4] (2014)	+	+	+	+	+	+	+
Khademi et al. [41] (2014)	+	+	+	+	+	+	+
Yoo et al. [42] (2014)	+	+	+	+	+	+	+
Zhang et al. [43] (2014)	+	+	+	+	+	+	+
Alobaid et al. [44] (2014)	+	+	+	+	+	+	+
Nagata et al. [45] (2014)	+	+	+	+	+	+	+
Kahler et al. [46] (2014)	+	+	+	+	+	+	+
Nagy et al. [47] (2014)	+	+	+	+	+	+	+
Tawfik et al. [48] (2013)	+	+	+	+	?	+	?
Zhu et al. [49] (2013)	+	+	+	+	+	?	+
Jadhav et al. [50] (2013)	+	+	+	+	+	+	+
Sönmez et al. [51] (2013)	+	+	+	+	+	+	+
Mc Tigue et al. [52] (2013)	+	+	+	+	+	+	+
Martin et al. [53] (2013)	+	+	+	+	+	+	+
Petrović et al. [54] (2013)	+	+	+	+	+	+	+
Dabbagh et al. (2012) [55]	+	+	+	+	+	+	+
Chen et al. [3] (2012)	+	+	+	+	+	+	+
Jeeruphan et al. [6] (2012)	+	+	+	+	+	+	+
Kim et al. [56] (2012)	+	+	+	+	+	+	+
Iwaya et al. [57] (2011)	+	+	+	+	+	+	+
Torabinejad and Turman [20] (2011)	+	+	+	+	+	+	+
Cehrelli et al. [16] (2011)	+	+	+	+	+	+	+
Yamauchi et al. [58] (2011)	+	+	+	+	+	+	+
Nosrat et al. [59] (2011)	+	+	+	+	+	+	+
Petrino et al. [60] (2010)	+	+	+	+	+	+	+
Zuong et al. [61] (2010)	+	+	+	+	+	+	+
Da Silva et al. [62] (2010)	+	+	+	+	+	+	+
Thomson and Kahler [63] (2010)	+	+	+	+	+	+	+
Reynolds et al. [22] (2009)	+	+	+	+	+	+	+
Ding et al. [64] (2009)	+	+	+	+	+	+	+
Bose et al. [65] (2009)	+	+	+	+	?	?	+

+ = low risk; ? = unclear risk; and − = high risk.

**Table 4:** Summary of all old systematic reviews in the scope of our systematic review.

Authors	Year	Number of studies used	Method summary	Main conclusions
Bucchi et al. [66]	2017	23 studies	The systematic review summaries and presents different clinical and animal studies performed. Only those articles published up to May 2016 were considered for review. Using 7 different databases (MEDLINE, Scopus, Cochrane library, SciELO, Google Scholar, Science Direct, and EMBASE), an electronic search was performed.	Most of the included studies did not follow a standard clinical protocol for regenerative endodontic therapy.

Antunes et al. [67]	2016	11 studies	A systematic review summarizes and presents original articles in the database Web of Science, PubMed, BVS (Medline, SciELO, Lilacs, and BBO), Scopus, and Cochrane. Only those articles published up to July 2014 were considered for review, and analysis of the papers published during this period took place based on previously established criteria, through the methodology of a systematic review.	Significant outcomes have appeared in the pulp revascularization, but several aspects remain unknown, such as the key factors of this repair, the type of tissue formed, and the long-term prognosis.

## Data Availability

The data used to support the findings of this study are included within the article.
